# Reaching internal consensus: Decision-making by transgender and plural people

**DOI:** 10.1371/journal.pone.0335714

**Published:** 2025-10-30

**Authors:** Silver Mckie, Sana Flynn, Christopher Wolf-Gould, Susan C. Turell, Matthew A. Adan, The Redwoods

**Affiliations:** 1 Department of Anthropology, Washington University in St Louis, St Louis, Missouri, United States of America; 2 Counseling Psychology PhD Program, College of Education, University of Houston, United States of America; 3 Gender Wellness Center, Bassett Healthcare, Oneonta, New York, United States of America; 4 Department of Medicine, Massachusetts General Hospital, Boston, Massachusetts, United States of America; Nanjing University of Aeronautics and Astronautics, CHINA

## Abstract

People who identify as both transgender and plural (more than one person sharing a body), including those with a diagnosis of Dissociative Identity Disorder, make decisions related to gender identity and presentation, and may engage with healthcare providers to receive gender affirming care. Internal decision-making by people experiencing plurality has not been studied extensively. Furthermore, the existing literature on plural decision-making does not address the intersection of transgender identity and the associated choices to be made about external gender expression or shared body modifications. Using a community-based participatory research design and a non-pathologizing lens, the research team interviewed 15 transgender and plural participants. Through thematic analysis, three themes were developed, describing the context of conflict, collective decision-making processes, and solutions that promoted harmony within plural systems. Plural participants were able to effectively navigate decision-making regarding harmful societal narratives about transgender identity, external gender presentation, and receiving gender-affirming medical care. Recommendations from the data serve to assist clinicians in understanding and supporting affirming, autonomous and informed decision-making by trans and plural clients.

## Introduction

The intersection between the transgender population and dissociative identity disorder (DID) appears infrequently in scientific literature, with very few publications addressing how this population approaches decision-making. In a review [1] of 37 articles from 2015–2020 addressing transgender mental health, only one study commented on dissociation in transgender populations. In a review [2] of five studies, only one of 577 transgender participants had a diagnosis of DID. Beyond infrequent case reports, a few studies [3–5] have found significant dissociation in the transgender population, while three articles [4–7] noted that dissociative symptoms decreased after patients underwent gender affirming hormonal or surgical treatment. Recently, Turell et al. [8] conducted a qualitative study investigating the intersecting experience of holding both transgender and plural identities, confirming that people who are transgender and plural have rich, embodied experiences of gender that manifest internally and externally.

The term ‘transgender’ broadly describes a population that experiences incongruence between their internal sense of gender identity and the gender assigned to them at birth, and it is important to recognize their identities as authentic [9]. Transgender people may be diagnosed with gender dysphoria, which “denotes persistent discomfort with one’s biological sex or assigned gender” [10 p.1]. Hormone replacement therapy or surgical procedures may be offered to alleviate gender dysphoria. There is an international movement away from a pathological framing of transgender identity, with the ICD-11 abandoning the classification of transgender people as mentally disordered [11].

Experiences of plurality familiar to clinicians include Dissociative Identity Disorder (DID) and Other Specified Dissociative Disorder (OSDD). They are characterized by “various simultaneously active and subjectively autonomous strands of experience that are rigidly and profoundly separated from each other in important ways, such as in memory, characteristic affects, behavior, self-image, body image, and thinking styles” [12 p.3]. These strands of experience are characterized as multiple identities, internal people, self-states, or ‘alters.’ When alters take control of the body, they can be described as ‘fronting,’ and exchange control in a process termed ‘switching’ [13]. The term ‘system’ describes the collection of these entities sharing a body, while the term ‘system member’ is a neutral term, equivalent to ‘alter,’ which describes one individual entity within a plural body [14–16]. Members of a system may have unique experiences of gender and salience of gender, perceived internal appearance, age or experiences of age, varied beliefs, memories, feelings, and thoughts [12,17]. Alternatively, those who view themselves as only one person or entity in a body are referred to as singlets [18,19].

Dissociative Identity Disorder has largely been explored through models which argue dissociative experiences of personhood primarily arise from traumatic experiences in childhood [20,21]. Much of the scientific literature pathologizes multiple identities as an indication of dysfunction, deficiency, and instability and recommends treatment to unify multiple identities into a singular identity [22,23]. While aligning with a pathological framework, the International Society for the Study of Trauma and Dissociation [24] also recognizes that a significant population of those with DID are unable or unwilling to unify into a singular self, instead working towards better communication and coordination among the various identities within the system. This outcome is fairly common, as Christiensen [25] found that 78% of 863 individuals that self-reported a diagnosis of DID (or otherwise identified as ‘multiple’ or ‘plural’) preferred to maintain a functional state of multiplicity.

A subset of scientific literature recognizes that experiences of multiplicity manifest in non-pathological presentations [26–29], and has found value in a holistic or non-pathological approach to dissociation [7,30–34]. A recent literature review by Eve, Heyes, & Parry [35] conceptualized a continuum of multiplicity experiences ranging from nonpathological multiplicity to clinical DID. They noted that professional ignorance of nonpathological or subclinical multiplicity resulted in the over-medicalization of participants’ experiences.

The present study uses the term ‘plurality,’ which emerged from the advocacy community of multiples, and recently has been incorporated into the scientific literature [8,16,18,36–41]. This newer, more inclusive term describes a broad range of pathological and non-pathological multiplicity, denoting those who have more than one person or entity sharing one body as a ‘plural system.’ [16]. While the term ‘plural’ includes those in clinical distress and diagnosed with a dissociative disorder (DID or OSDD), authors have noted that many identifying with plurality found ways to live well with dissociation [19,35] or did not experience distress from plural experiences [38,42].

Members of the plural community may present to health care providers requesting medical or surgical care for gender dysphoria. Pathologizing models of DID raise concerns about the decision-making capacities of those experiencing any form of multiplicity, with some clinicians calling for a years-long process of unification before the patient makes significant medical decisions such as those involved in transition care [43,44]. However, there are various populations that may want to pursue transition-related care while remaining plural: people with DID who choose not to unify, clinical populations intending to unify who require time-sensitive transition care prior to completing DID-related treatment, and a broader population experiencing sub-clinical or non-pathological plurality. As transition care can be lifesaving in many cases [45,46], it is crucial that the clinical community understands how to navigate patient-affirming decision-making and informed consent procedures relating to transition care with plural patients.

Both transgender people and those experiencing dissociative symptoms face multiple barriers when accessing healthcare [35,47], and may face implicit bias from providers which contributes to health disparities and non-affirming treatment [48]. Such barriers include a lack of trained providers, time and financial constraints, provider misconceptions, discrimination while receiving healthcare, and disbelief expressed by providers [49,50]. Turell et al. [8] found that clients who were both transgender and plural faced barriers to care when providers had limited knowledge about plurality, especially when providers mistakenly assumed that the client’s plural identity invalidated their transgender identity or capacity for informed consent.

People who are plural are able to develop a deep, rich, and embodied knowledge of their gender, which informs decisions to seek gender-affirming care in those who also identify as transgender [8]. Plurality should not preclude transgender patients from making informed decisions regarding gender affirming care [8,38], as clients with dissociation have demonstrated intentionality when pursuing transition care and can live peacefully with their decisions [51]. In regards to medical decision-making processes in people with DID, Brennan [52] concluded that the client’s autonomy and self-governance should be prioritized whenever possible, and that only “severe value-incongruence and conflict” [p.81] between system members indicates an inability to make a collective decision.

Assessing the subjective experience of conflict in DID, Marais, Bezuidenhout, & Krüger [53] found that conflict arose when system members experienced incompatible feelings, thoughts or behaviors. This is consistent with previous literature on conflict in DID [54,55]. Marais, Bezuidenhout, & Krüger [53] identified six common sources of conflict between system members: withholding information from other system members, aberrant behavior that other system members did not condone, differing patterns of emotional expression, incompatible goals, conflicting value systems, and control over the physical body. Conflict was exacerbated when participants lacked awareness of their multiplicity.

Many authors [12,13,24,33,55–59] agree that resolving conflict in DID involves accepting all system members and internally committing to cooperating as a functional team. Putnam [55] notes that many people with DID already have a foundation for a cooperative decision-making process, which can be supported by the therapist [12,57,60]. The subject of the decision may involve more superficial activities, such as the completion of daily tasks [58], or deeply emotional matters such as confronting fears, navigating relationships, and identifying life goals [37,56]. Decision-making should be a fair, impartial, and democratic process focused on the overall well-being of the system [55]. However, negotiating conflict is often an ongoing process for those experiencing either pathological or non-pathological multiplicity [35].

Clinical examples describe a variety of internal strategies regarding decision-making processes. Most frequently, multiples held group discussions and allowed all system members a vote on potential outcomes [13,55]. Specific system members may be designated to facilitate group discussions [17,56] or elected to a decision-making council if group discussions are not feasible [55]. Henkin [33] describes the outcome of decision-making processes as a “consensus: a weight of support from the people inside agreeing with what the system is doing, while those parts that don’t agree, agree to support the team and not to get in the way” [p.177].

Encouraging system members to have empathy for one another while discussing difficult matters helps system members develop a sense of safety with one another and reach an agreeable conclusion [12,56]. Putnam [55] notes that system members who have previously acted out of selfishness begin to cooperate with the group as they are given the space to voice their needs, be acknowledged with empathy, and have those needs met through the decision-making process.

The present study serves to address related gaps in the scientific literature regarding conflict resolution, decision-making [53], and experiences of medical care for plural clients [35,50]. While there are numerous clinical resources that mention cooperation and conflict resolution in DID, few give detailed descriptions of collaborative decision-making between members of a plural system. Furthermore, there is a lack of literature regarding the assessment of reasoned decision-making and consent in plural persons undergoing medical procedures unrelated to gender-affirming care [61,62]. The present study documents internal decision-making processes from the perspective of transgender plural persons themselves and lends depth to continuing conversations about consent, decision-making, and medical care of plurals.

## Methods

The data for this study was collected to explore the broad intersection of transgender and plural experience [8], and underwent a secondary analysis to examine internal decision-making strategies for transgender plural people. Thematic analysis was performed on interview transcripts of 15 participants who self-identified as both transgender and plural. Study design drew upon Charmaz’s [63] constructivist grounded theory and Braun and Clarke’s [64,65] reflexive thematic analysis. Incorporating a community-based participatory research model, half of the research team identified as trans and plural. The aim of the original interview questions was to explore the interface of participants’ gender identities and plurality.

### Ethics statement

The study was approved by The Mary Imogene Bassett Hospital Institutional Review Board, which oversaw the collection of data, preliminary analysis, and current analysis. Participants were read a consent form by the researchers upon enrollment in the study, and gave verbal consent. Verbal consent was approved by the Institutional Review Board and utilized for efficacy as interviews were conducted remotely.

Interviews were conducted over a HIPPA-compliant video platform. Names, identifying information, and Protected Health Information (PHI) were removed from the recordings before transcription. Demographic information was stored separately from both the recordings and interview transcripts. Interview transcripts were de-identified, and the original recordings were destroyed. Only original research members performed the secondary analysis.

### Procedure

Recruitment was done through community networks of trans and plural people, identified by members of the research team. A solicitation for study participation was distributed by community outreach to specific individuals and to online communities known to include a high number of trans and plural members from October 15th, 2020, to December 26th, 2020. Both the outreach to personal individuals and the online solicitations led to successful recruitment. The first solicitation led to a response by 14 people who self-identified as both transgender and plural, 10 of whom ultimately consented verbally to participate in the study.

Interviews were held remotely on a secure video platform, conducted by either Susan Turell Ph.D or Christopher Wolf-Gould MD. Gift cards ($50 USD) were distributed to participants after completion of interviews. Recordings of the interviews were then transcribed and deidentified prior to thematic analysis, following the six stages outlined by Braun and Clarke [64,65]. Ten initial interviews were collected for analysis. Member checking was then done to enhance credibility. The original 10 participants were invited to a focus group to provide feedback on preliminary findings, of whom five agreed to attend. This discussion resulted in identification of further areas for exploration and led to the formulation of five new interview questions (S1 Fig). Five additional interviews including these new questions were conducted from April 28th, 2021, to May 22nd, 2021. The data from all 15 de-identified interviews were used in the current analysis.

The sometimes-dissociative nature of living as a system impacted this study and interfered with the interview process at times. Some participant systems switched between members during the interview, either inadvertently or on purpose. Verbal consent was utilized to ensure consent from the system member fronting at the time of the interview. It was not possible to interview all members in each system – in order to include perspectives of non-fronting members, the system members fronting during the interview reported the perspectives of other system members. Dissociation occasionally led to difficulty remembering interview times, leading to rescheduling of interviews. The interviewer offered breaks, the freedom to end the interview at any time, and therapist referrals; some participants needed a break during the interview process to ground themselves but all completed the interview.

### Data analysis

Primary analysis of the 15 transcripts was done using thematic analysis; a line-by-line analysis was used to generate open codes, which were then aggregated to construct overarching parent codes and a code book. Reflexive thematic analysis was done, using six phases described by Braun and Clarke [64,65]. The individual transcripts were divided among three authors, a mixed group of plural and singlet researchers, who coded their subset of transcripts independently. Afterwards, the three researchers reviewed all codes as a group to ensure consensus and discussed specific lines that were difficult to code. Although intercoder reliability was not quantified, these collaborative discussions were held until consensus was reached for all codes in order to insure trustworthiness of data analysis. The code book has sequential iterations, allowing examination and review of the thematic analysis over time. The final results were reviewed by the larger, 6-person research team prior to manuscript completion. Researchers’ lived experience, as members of the trans plural community, clinicians, or both, informed the analytic process.

The interviews were not tailored specifically to problem-solving processes, instead focusing broadly on the intersection of trans and plural experiences. For this paper, the authors focused on an emergent topic generated by review of the source material: the problems, decision-making processes and resolutions reported by participants. The authors defined ‘problems’ as situations which negatively impacted their functioning, caused internal conflict among system members, or prompted participants to make decisions. ‘Solutions’ were defined as resolutions to problems that were discussed, worked towards, or achieved by participants. A ‘process’ was defined as a way to reach the solution, and could be planned by participants or may have naturally emerged. Both processes and solutions could be unproductive or unsuccessful. After parent codes were generated, problems were categorized into those where the participant system had found a clear solution versus those without. Commonalities in types of problems and decision-making processes were identified, leading to the development of themes and subthemes.

## Results

### Participants

Demographic information is presented in Table 1; The ages of the participant’s bodies ranged from 18–38. Body ethnicity/race included 60% systems of color (African American n = 2, Asian n = 2, mixed ethnicity n = 5), and 40% white only. Three were assigned male at birth (20%), ten were assigned female at birth (67%), one identified as intersex (7%), and one declined to answer.

**Table 1:** Participant Demographic Characteristics.

System Pseudonym	Self-reported number of system members	Gender Identities	Age of Body	Gender assigned at birth of body	Ethnic/Racial identity of body
Elm*	5	Queer, trans, non-binary, gender neutral	19	Female	White
Sycamore*	4	Non-binary, femme	22	Female	White
**Number** Seven*	38	Non-binary, cis-girls, masculine, sirens, agender, pangender, transfeminine	27	Female	White
**Willow**	18	Non-binary, agender, X-Jendaa, cis-male, cis-female	21	Intersex	Native American, Asian
**Maple**	Unlimited	Non-binary	23	Female	African American
Lennon*	16	Genderfluid, gender queer	19	Female	White
Finley*	12	Non-binary,other terms	18	Female	East Asian
**Ash**	230	Nonbinary, male, female, agender, genderfluid	21	Female	White
**Denver**	2,000	Man, woman, others	22	Female	Native American, White
**Landry**	20	Transwoman	18	Male	Native American, White, Irish
**Parker**	4	Transfeminine	38	Male	Asian
**Jesse**	11	Genderfluid	37	Female	Asian, White
**Kerry**	8	No Label	32	Declined to Say	African American
**Chantal**	21	Transfeminine	20	Male	White
**Orange**	12	Transman	21	Female	African American, White

*Focus group participant.

Participants described their internal entities using words such as alters, headmates, system mates, system members, brainmates, entities, and spirits. The authors use the term ‘system members’ in this report, as it is a neutral term regarding the pathologization of plurality used by various groups within DID, plural, and multiple communities [14,15,66]. The reported number of system members ranged from four to unlimited. Language used to describe plurality included system, plural, DID (dissociative identity disorder), OSDD (other specified dissociative disorder), multiplicity, polyfragmented and circle, with plural the most commonly used.

The average time of awareness of plurality was six years with a range of less than one year to twenty-two years. Four of the participants reported having a formal DID diagnosis. The length of time identifying as transgender ranged from one to thirty two years, with an average of nine years. Ten participants identified as transgender prior to plurality, three systems realized their plurality prior to identifying as transgender, and two participants realized both identities at the same time.

### Thematic analysis

Three overarching themes were generated from the data: (1) Framing the Situation: Conflict as a Fact of Life, (2) Collective Operation: The Processes, (3) Outcomes that promote system-wide harmony (Table 2).

**Table 2:** Thematic Analysis Themes and Subthemes.

Themes	Subthemes
**Framing the situation: conflict as a fact of life**	Gender identity, expression, and dysphoria Harmful societal narratives Understanding member individuality and group needs as precursor for problem solving
**Collective operation: the processes**	Communication: the exchange of information between system members Collaboration: the act of working together Power dynamics affecting collaboration Non-linear and messy processes
**Outcomes that promote system-wide harmony**	You do you – as long as it doesn’t negatively impact me Achieving equity by balancing system members’ incompatible needs Internal support: learning to love each other External presentation: how other people see us Pride: finding our authentic intersecting selves

### Theme 1. Framing the situation: Conflict as a fact of life

People who identify as plural have multiple conscious system members within a body, all with their own wants, needs, and opinions. Any group of external people are certain to encounter conflict at some point; similarly, several entities in a body are bound to eventually have incompatible desires about what to do with a limited amount of time and a shared body. The participants grappled with issues that significantly impacted certain system members or the system as a whole. Participants needed to make decisions in order to resolve both internal and external conflicts.

### 1.1. Gender identity, expression, and dysphoria

Participants struggled with having conflicting experiences of dysphoria between system members, such as a female system member feeling dysphoria about masculine features concurrent with a male system member feeling dysphoric about feminine features. They often had to make decisions about how they wanted to embody their gender identity. Such decisions involved outward gender expression, pronoun use, and physical transition of the body.

“[X system member] will decide to dress in the morning and just do something that is comfortable to her. And I find that, if for whatever reason, she isn’t [fronting]… then I feel really, really dysphoric in most of the clothes that she wears.” (Kerry System)

“I would experience complete dysphoria in both directions simultaneously… “Oh my gosh, I miss my long hair. I want my long hair back. I want to wear more feminine clothing,” …and then simultaneously and just as strong would be, “Oh my gosh, my voice is too high and I look too much like a female and like hips and legs and how do I walk like a male, like what the heck, I can’t do this. This is wrong.” And, I would just sit there like, “What the hell is going on?” (Lennon System)

### 1.2. Harmful societal narratives

Many participants grappled with pervasive societal expectations about normative identity. One participant noted that “society expects you to not only be cis but also [to] be singular” (Finley System). Challenging societal norms by existing authentically as transgender or plural may be dangerous, and participants needed to determine as a group whether or not they felt safe to challenge societal norms and effectively field instances of discrimination before making related decisions.

“When we’re both [fronting] we’ll ask people to use [X name] and [Y name] interchangeably… Not a lot of people actually respect that. They’ll be like, ‘oh, that’s too complicated. I’m just going to pick one.’” (Lennon System)

In addition to this singlet normativity and cis normativity, participants reported binary transgender normativity – expectations that transgender people should all transition fully to the other binary gender, and disbelief in non-binary identities – as well as systemic racism when participant system had a cultural identity other than a White Western identity. Many participants grappled with harmful self-expectations internalized from the dominant culture, such as internalized transphobia, which served as a source of internal conflict among system members and caused internal struggle to accept their own identities as either trans or plural.

“If someone misgenders me, I don’t generally correct people that I don’t know, because it just becomes this whole thing… I’m so tired… people don’t think plurality is real and they don’t know what it is… people saying, “that’s not real” [happens] with plurality [and] with being trans, especially non-binary.” (Elm System)

“We have a cultural gender within our system. And people hear that and they think it’s not a real thing or they can’t grasp it. And we have to kind of Westernize it for them.” (Willow System)

“The whole entire idea that’s fed towards a lot of queer people is that… you have to suffer in order to be trans, and we felt like we didn’t suffer enough… It took us a lot of unlearning and reading through other trans and non-binary people’s accounts to slowly start chipping away that internalized transphobia and say that, number one, trans doesn’t equal dysphoria and I can be whoever I want.” (Finley System)

Participants reported that some medical providers believed that one could not know their gender while experiencing plurality, and therefore might withhold transition-related medical care from their plural clients until they underwent therapy to form a single, non-plural identity. Therefore, some participants expressed concern that disclosing plurality would limit their access to gender affirming care.

“The psychiatrist that we saw… wasn’t comfortable with us allowing us to transition if there were [system members] who were not male. So, she wanted us to integrate [into one person]… it’s not like she forced us, like obviously, we agreed to it, but it’s because we felt that we had to do that to transition…. eventually, [system members] just started peeling off again and unintegrated, de-integrated spontaneously. So, that was horrible and traumatic and made us not want to do therapy for years after that. I feel like that’s probably the most traumatic experience of how medical and psychiatric stigma against plural people has harmed us.” (Jesse System)

### 1.3. Understanding member individuality and group needs as a precursor for problem solving

In order to approach decision making in a coherent fashion, participants needed to understand both themselves as individuals and as a group existing in the same body. Many participants found it difficult to make decisions before discovering and understanding internal processes related to their plurality. They first needed to identify their own system members, and distinguish each member’s unique inputs. When unaware of their plural identity, strife was often misinterpreted as unresolved singlet inner conflict.

“Even after I found out I was plural, I had more identity issues come up because of realizing where everyone’s emotions were coming from… trying to pick apart which one belongs to me and which one belongs to who was also a struggle. And that was really hard to figure out for the first like year or so… but it’s way better than it used to be three years ago.” (Orange System)

In some circumstances, decisions about medical transition had been made before the participants discovered their plurality, which precluded input from various system members. These system members had to accept these decisions – which included transition steps already taken – and find ways to be comfortable with the body regardless of its current form.

Some participants noted that the group needed to shift their focus towards their internal satisfaction rather than external expectations; for example, expressing gender in ways that were meaningful and joyful rather than aiming for a societally-endorsed gender ideal or simply avoiding dysphoria.

“I try to live my life avoiding dysphoria, and I realized that that wasn’t working out… It was more important, for me, to look at euphoria and what made me happy, what made us happy. So, we try to dress and act and treat our body the way that makes us happy.” (Number Seven System)

Stress effects on emotional health – such as recurring symptoms of traumatic stress, coping with the impact of physical disability or mental illness, and feeling unsafe – complicated decision-making processes or served as a compounding source of internal conflict. Understanding the impact of stress allowed participants to access support from other system members or external people, which facilitated communication and collaborative decision-making within the system.

“She [system member] got triggered out [inadvertently fronting]… and I couldn’t communicate with her…. Then the other day, she got triggered out, and my boyfriend held us for like five minutes and we were fine” (Elm).

### Theme 2. Collective operation: The processes

When making lasting or impactful decisions, our participants reported a need for collective operation. System members worked together as a group to find solutions that would best suit everyone and promote harmony within the system.

### 2.1. Communication: The exchange of information between system members

Each individual system member brought a unique perspective to the decision-making table, shaped by their individual wants, needs, and experiences. Potential outcomes affected each system member differently. This was especially salient in regards to gender dysphoria and transition goals, attachments to names and objects, and capacity to handle discrimination.

Participants had diverse processes in developing internal communication; some systems put significant effort into circumventing dissociative barriers, while others were able to communicate freely with minimal effort. When unable to communicate internally, participants utilized various communication techniques, such as fluidly writing out thoughts or leaving notes to each other in a journal. All participants were eventually able to find some method to foster internal communication.

“We started with leaving notes in a journal, handwritten stuff. It started off with questions for each other… “What is your name? How old are you? Tell me about yourself.” So that we could kind of start getting a handle on who all we were. At this point, we use Discord [messaging platform] … [to] help negotiate things if need be, or leave notes.” (Denver System)

During the decision-making process, system members informally communicated among each other to learn about each other’s unique desires, needs, opinions, and experiences. Participants acknowledged and honored opinions that differed from the majority. They incorporated lessons from other system members into their own worldviews. Sometimes, spending time together helped system members better understand one another.

“If you’re changing the body’s name, definitely, you’ve got to tell everybody and talk about it… Like how are you going to cope if someone is really attached to [the old] name?... You have to sort of sift through everything, but that comes from listening and being compassionate and even knowing that she’s attached to that name in the first place. You know, this is kind of obvious, but it takes time.” (Sycamore System)

“I love that [being plural] can provide so much insight. I didn’t understand… how do you have no gender? I don’t get it. But like I said, one of our Littles [child system member] identifies as agender, and I have a close relationship with that Little. And if we are co-front or co-conscious at all, I can kind of get a little more insight on what that feels like.” (Number Seven System)

### 2.2. Collaboration: The act of working together

Given the diversity of plural experiences, the process to reach consensus was unique for each system. For some participants, the process was brief and easy, while others described it as an intensive or continuous process. When faced with difficult decisions, participants held multiple meetings between system members. These meetings were an intentionally created space where conflicting opinions and experiences could be talked through between system members. Some participant systems held fluid, free-form discussions, while others held structured meetings where certain system members assumed specific roles to facilitate discussions.

“Just because I’m a system doesn’t mean I can’t make up my own mind. We usually have very intensive meetings before we come to a decision about the body… We’ve got managers of different areas who are responsible to go around and ask the people in their area, “So, what do you think about this? What do you think about this?” And then, all the managers come together when they can and share the data that they collected.” (Ash System)

Internal engagement in decision-making varied. For some participants, all system members were deeply involved in finding an agreeable result. For others, not every system member wanted or was able to be involved. Inclusion mattered, and participants expressed dedication to include as many members of their system in the decision-making process as possible, regardless of the format.

“It’s not like a “one vote, one member” kind of thing. It just means we discuss things until we come to some kind of agreement, even if it is a compromise, even if it’s something that some people aren’t completely happy with, it’s something that they can accept.” (Jesse System)

### 2.3. Power dynamics affecting collaboration

Some systems gave more decision-making power to members who were disproportionately impacted by the outcome of the decision, either by being consistently active in the external life or being uniquely connected to the issue at hand. This was especially relevant when discussing physical transition, as the quality of life of system members who experienced intense levels of dysphoria could significantly change based on the system’s transition goals. Systems considered their own internal power dynamics equitable when they reflected what would be best for the group’s functioning as a whole, and when all system members consented to a given arrangement.

There was a complicated interplay between system members’ gender identities, experiences of gender dysphoria, and frequency of fronting. Some participants mentioned that fronting frequently made them feel more connected to the shared body. In turn, some participants’ system members who felt more connected to the body tended to identify as transgender and experienced bodily gender dysphoria, while sometimes those who infrequently fronted viewed the shared body as unreflective of their self-conception and did not experience gender dysphoria. However, fronting frequently wasn’t a requirement for feeling connected to the body. Some participants noted that system members may choose not to front in order to avoid gender dysphoria; they fronted more often after pursuing agreed-upon physical transition, which relieved their dysphoria.

“Oftentimes the people who front more often have more say in terms of what the body actually transitions to be. There are system members that, out of the twenty years that this body has been alive, probably have only fronted like twice. So, their decision to have the body transition in a certain way is less [of a] priority because they don’t even have to live with their own decision.” (Chantal System)

Some participants had a history of inequitable internal power dynamics within their system, when one (or a few) system members would exercise control over decision-making to exclude certain members from participating in group discussions. Several participant systems reported periods where they mistakenly believed only one system member was a “real person,” and deprioritized the needs of all other system members. All participants were able to resolve these inequitable power dynamics either by practicing compassion and inclusivity, holding interventions to discuss exclusionary dynamics, or through individual system members self-recognizing and healing harmful patterns of behavior. Similarly, a few participants’ system members had to work through internal biases, such as male system members with internalized toxic masculinity disregarding female system members, before they were able to collaborate.

“You really shouldn’t say, “I’m the host, so I get to ignore everyone else”... The host spends the most time out so sometimes their opinion gets to have a little bit more weight, but… no one alter should say, “this is final” without talking to everyone else.” (Finley System)

“They were not what I would call good consensus-making processes… no one’s desires were important, except for [X system member]’s… This is his life and his body that we’re just passengers in. It’s been almost 15 years since that time… [these were] frankly kind of awful conclusions about personhood and power… We have restructured the way that we do front management and cross-talk in such a way that… [X’s wishes] no longer have so much power over my system.” (Jesse System)

“Because I front the most, I’m sometimes considered the “real one,”… it’s almost like an internalized belief, where I know it’s not true, but I have to fight to be able to listen and not have [my] own agenda at work.” (Sycamore System)

“I have to step back and remember I have been socialized by a society that is misogynistic, that is patriarchal, and I have to watch how I think about my female and non-binary headmates so that I’m not infantilizing them or looking at them as less capable… If I’m thinking, “I mean well. I want to protect them,” I have to think, “well, where is that coming from?”... I suspect that it’s complicated for my other male-identified headmates as well. It’s something we’ve talked about. It’s something we continue to talk about.” (Jesse System)

### 2.4. Non-linear and messy processes

In contrast to conflicted yet respectful discussions, some participants reported periods of discord, where system members refused to engage with any collective process and acted out without consulting anyone else in the system. Examples included disregard for others, neglect of prior agreements, and fighting among each other when problems arose. Participants observed that refusing to work together not only stalled the decision process but caused harm to system members and the system as a whole. However, nearly all participants mentioned that discord could be resolved through persistent compassion and care towards other system members, emphasizing a shared life and the necessity of working together.

“At first, it was very rough. People would suddenly change something without talking to anyone. You know, someone would shave our head, and alters with long hair inside would be very upset. And, you know, “What am I supposed to do with this bald head now?” … Someone would throw away all of our masculine clothes and buy a bunch of feminine clothes or the other way around. It was kind of a lot of, “I’m just going to do what I want. I don’t care how it affects the others” at first.” (Denver System)

The decision-making process was not always linear. Some participants described a constant push and pull between internal opinions; sometimes the system would agree on a decision, only for one member to change their mind and prompt the entire group to re-evaluate their options. Sometimes, system members insisted that their specific needs be met or vetoed group decisions they strongly disagreed with.

“There was a lot of back and forth. There were a lot of times where we thought we had come up with something that we were all happy with. And decided or changed our mind in the end, realiz[ed] that some people were heard a little bit more than they were really entitled to.” (Kerry System)

“A lot of the guys just want to be done with it, just get top surgery and have it be over with. But there’s girls in our system who say, “Well we could just get a reduction and still have a little bit but not a lot. And, that way we’re kind of balancing my needs with yours…. And, guys saying, “Well I want to walk around shirtless… and I can’t do that if we don’t get top surgery.” And, just back and forth on “well, let’s compromise” and “I don’t want to compromise.” (Denver System)

When needed, participants delayed decisions, asked a trusted external person for advice, or continued negotiations despite internal resistance. Participants noted that when a system member refused to compromise, slowing down and listening to the dissenting system member with compassion was the best course of action.

“If there is going to be a drastic change… [and] if there’s strong disagreement, then we just don’t do it regardless of how strong other people feel about it positively.” (Denver System)

If there was no easy resolution, accepting that plural experience comes with some inevitable messiness allowed participants to come to terms with continuing disagreement. Despite obstacles, participants re-prioritized collective wellbeing and worked together to find the best available conclusion that could maximize meeting needs across the system, even if some people were left unsatisfied.

“I can say this statement: it’s messy. And, that applies to both being trans on its own and being plural on its own. So, when you put it together, oh my god, it’s so messy! And, that can freak people out… I know, for a long time, we wanted everything to sort of be in a box. I’m like, “Oh, I want you to be this and you to be that.” So, I guess, [it’s important] to know that it’s messy, and that’s OK. And, the messiness is changing.” (Sycamore System)

### Theme 3. Outcomes that promote system-wide harmony

Participants found different routes to harmonious resolutions between system members based on the context of the problems they faced and the impact they had on different system members. Many participants found a deep sense of satisfaction and acceptance when they were able to find a resolution.

### 3.1. You do you, as long as it doesn’t negatively impact me

In some situations, conflict could be avoided by allowing individual system members to pursue their unique wants and needs, as long as their actions did not negatively affect others in the body. Individual members defined their identities for themselves, independent of other system members or the body’s assigned identities.

“Each of us present however the heck we want… [X system member] identifies as genderqueer, and [Y system member] uses demiboy…. We use [Z name] more when we’re blended together than when we’re just both there. When we’re both there we’ll ask people to use [X name] and [Y name] interchangeably.” (Lennon System)

“We see ourselves as individuals and not just a part of the overall collective… For the cis-identified system members, that is their identity alone and they hold that within themselves, even though the body might have a different history.” (Chantal System)

Ownership of clothing or accessories that belonged to individual system members, as well as expressions of individual members’ gender identity through makeup or hairstyle, was meaningful and helped system members feel seen. Day-to-day decisions about gender expression which did not involve lasting change – “small things, like “what am I going to wear today?”” (Finley System) – did not require consulting with others in their system; whoever was fronting at the time decided in the moment.

“Everybody can choose something. The girls, they love painting and doing arts and crafts and stuff, so that was something that they would do to help them mentally. And, us guys, we like… playing basketball, going outside and doing some random stuff, we do stupid stuff! That was kind of how we leveled out. Everybody got to do what they wanted to.” (Maple System)

“We have a lot of clothes, so some days the guys would pick our clothes for the day. And then, some days the girls would pick their clothes for the day. Or some days [X system member] and his non-binary self would make his way up here and pick whatever, meaning we probably end up wearing a SpongeBob T-shirt and his overalls.” (Maple System)

Many participants arrived at such a place of flexibility easily, but a few participants expressed difficulty accepting individualized expression. In some instances, a system member wanted to control how the body presented even when not fronting, and attempted to restrict the gender presentation of other system members. To become comfortable with other system members presenting differently, these systems had to resolve inequitable internal power dynamics as discussed above and recognize that the body did not solely represent a single system member.

“We’ve had a lot of arguments about [how] our masculine alters are afraid of the body being seen as female at all, even if they’re not fronting. And so, we’ve had to have a lot of discussions about that... We can, you know, put limitations on it and put rules around it, but we can’t completely stop [female system members] from being comfortable with themselves.” (Denver System)

### 3.2. Achieving equity by balancing system members’ incompatible needs

Sometimes, internal collaboration efforts to support individual expression could not resolve system members’ incompatible needs. In these situations, participants chose to compromise or agreed to prioritize the needs of specific system members.

Participants might engage in reciprocal compromise to find an acceptable middle ground between multiple system members on an issue. Each system member would have some aspects of the solution they liked, and some aspects they didn’t like. In one case when there wasn’t an easy middle ground, a participant system chose to make up for uneven outcomes by granting favors or additional decision-making power on an upcoming issue to those who felt dissatisfied or did not benefit from the outcome. Most of the time, participants felt successful in finding a decision that they were comfortable with and could all agree upon.

“Every day is a compromise. And ultimately, it just boils down to understanding that, you know, these situations are going to happen and we’re just going to have to deal. There aren’t really great alternatives… we might try to find something that is, if not ideal for everyone, at least acceptable.” (Kerry System)

“If we bought something for [the female system members] on one trip, the next trip we would do would be something bought for the [male system members].” (Maple System)

“I also want to express that just because overall the body is transitioning to look more feminine rather than [looking] androgy[nous] or even masculine, it doesn’t necessarily mean that [masculine system member’s] wishes of gender expression [are] put all the way on the back burner… for our more masculine presenting system members, in the future, we want to get a binder for them.” (Chantal System)

“The girls worked really hard to make sure that us guys got what we needed and wanted. But now we’re trying to return the favor to them because they did so much for us and we appreciate them.” (Maple System)

Other times, participant systems decided as a group to prioritize the needs of system members with severe dysphoria which impeded daily functioning. When other system members consensually put aside their desires, they acknowledged the significance of the situation and the depth of the affected system member’s needs. Even if the agreement did not benefit each member of the system individually, group consensus often reflected what was best for the system as a whole with consent from those who did not benefit.

One participant discussed how their [female] system members prioritized [masculine] system members’ dysphoria when undergoing masculinizing top surgery:

“[Y system member] doesn’t feel like the dysphoria for her was bad enough to be worth making the other people in the system deal with having dysphoria in the opposite direction… [X’s dysphoria] was very significant and very disruptive to us because he did a lot of our actual working at the time, and a lot of our social interfacing.” (Jesse System)

Preemptively, some participants established system-wide rules, which often dictated their behavior around external relationships, expression, or openness about identities. These rules helped keep the system safe and comfortable, and breaking these rules was a source of infighting.

“There’s the agreement that we don’t use she/her terms…. sometimes people [system members] don’t follow the rules when we say, “hey, no. Don’t do that. Don’t tell them that [we use she/her pronouns].” We try to keep it [use of they/them pronouns] constant because it’s easier that way.” (Maple System)

### 3.3. Internal support: learning to love each other

Creating a healthy internal culture of care and support promoted holistic living and mutually benefited all members of the system. System members built relationships among each other over time, which in turn furthered their ability to understand other system members’ perspectives and accept each other’s differences. Strong internal relationships allowed system members to anticipate each other’s needs and offer advice, emotional support, and friendly gestures to each other during periods of hardship, which eased potentially stressful situations and prevented conflicts.

Participants actively sought to learn and anticipate the needs of other system members. They bought affirming or wanted objects and prepared comforting environments for other system members when they had a bad day. During difficult or stressful situations, system members would empathize with each other or offer advice; sometimes just the internal presence of other system members helped participants get through moments of hardship.

Drawing on personal experiences, one participant described how to help system members feel safe and comfortable:

“I think definitely getting to know who you’re talking to beforehand to know what is needed would be good… being like, “hey, do you like candles? I’ll buy one of those little battery-powered ones and put it here. Do you like flowers? I’ll get some flowers. Do you like stuffed animals? I’ll put stuffed animals out.” Physical things like that would be good [to build trust and safety].” (Willow System)

“Part of the medical interventions involved when being transfeminine for us are uncomfortable physically, emotionally, or both. … it’s usually been me in the chair, so it’s really nice for me to have company and to have somebody there with me even when we can’t have another body-person, just to have somebody who can kind of emotionally hold my hand.” (Parker System)

Participants described how certain system members had unique skills in situations such as work performance, navigation of mental health crises, and socialization. System members relied on each other’s strengths, allowing others in the system to step forward to handle difficult situations. In multiple cases, system members guided each other’s growth in understanding their gender identity and processing harmful internalized biases.

“But there are times when [being plural] is kind of helpful or beneficial. I have headmates who manage mania and I guess probably also depression better than I do… There are a lot of times that I probably would have gotten myself injured or harmed, or possibly even killed, if I did not have people in my head who are able to influence me in various ways, or just straight up kick me out of front when I’m not able to handle things. I have headmates who do… a lot of other tasks better than I do, who are more organized than I am.. having people who have different skillsets and different preferences or interests than you, is sometimes kind of handy.” (Jesse System)

“But [X system member] actually helped me with my body image, not just with embracing the way my body was… he helped me become proud of that… I used to struggle with being dark and like my racial identity. And [X] actually helped me a lot with my skin and my complexion…. He helped me deconstruct [internalized racism]. And he made me feel proud of having dark skin. And he said, ‘it’s beautiful. I love it. And you should embrace it.’” (Monroe System)

“Your confusion [about gender] and then learning more about yourself, it’s going to help your system. Because [other system members are] looking up to you. And so, them seeing you go through this confusion… may make them feel, “OK. It’s OK for me to feel this way too”” (Maple System).

### 3.4. External presentation: How other people see us

All participating systems were made up of system members who shared a body and a life. Therefore, system members had to agree on how they wanted to present themselves to the external world. Many participants felt unable to be open about their plurality to others, and therefore had to make decisions related to presenting as a single person.

Some participants held a shared communal identity when the system as a whole was situated within an identity group, such as ‘transgender’ or ‘queer.’ This sense of shared identity was organically experienced within the system, and included all system members while simultaneously leaving room for their individual identities.

“A lot of the frequent fronters are more feminine-leaning or non-binary or other things along those lines. And that is one of the reasons why we consider ourselves trans. Due to us discovering our trans identity back in middle school, I feel like we’ve held on to that label for such a long time that it has pretty much solidified in our mind. And then later on, whenever we found out – or the host at the time, I guess – found out that they were part of a system, it was like the trans thing is already solidified so now it’s just adding upon the understanding of plurality… even though collectively we identify as trans, we still have those sys[tem]-members who identify as being cis [men and women]… within a plural system.” (Chantal System)

Other participants purposefully constructed a singletsona presentation: a singular artificial identity assumed by the entire system to smooth and simplify interactions in the external world. Participants often created them for safety, using them when they were unable to be open about their plural identity. In contrast to communal identities which arose organically, singletsona identities were described as a collaborative creation; all system members who front agreed to a shared name, pronouns, gender identity, and particular gender presentation in order to smooth relationships with external friends, family, and acquaintances.

One participant system discussed their genderfluid singletsona identity:

“We’re not going to be out as plural to most people we interact with… We decided that a genderfluid or genderqueer [singletsona] identity was the best way to make sure that no one’s gender identity was erased, even when we are pretending to be one person.” (Jesse System)

Participants also needed to make compromises about how the physical body looked. When considering physical traits that could not be changed quickly – such as physical transition, piercings, and haircuts – some participants agreed upon a collective external gender expression that allowed flexibility for system members to present however they wanted.

One participant system discussed their decision to present their body androgynously:

“[Masculine system members] are fine with [transitioning] since they can still make us look more masculine as long as we’re still very androgynous.” (Landry System)

Another participant mentioned; “We’ve intentionally picked a hairstyle that works regardless [of who fronts]. We’ve had the same hairstyle since we were ten.” (Kerry System)

In a few cases, participants decided physical transition was the best course of action, even if it caused some system members conflicting gender dysphoria. To make these system members comfortable, participants maintained options for contrasting gender expression after physical transition, such as wearing feminine clothes or keeping their hair long after masculinizing surgery.

“Well, how does it work for the female system mates now that you’ve had, you know, top surgery? [mastectomy] … they knew that, they sort of made a sacrifice there, right?... But at the same time, you know, I feel more comfortable to wear dresses and stuff and other more feminine clothes. So, now that means [X system member] can wear that stuff too.” (Sycamore System)

### 3.5. Pride: Finding our authentic intersecting selves

When able, participants benefited greatly from proudly embracing their transgender and plural identities. Many found that accepting their plurality made it much easier to understand their other identities, smoothed the decision-making process, and allowed them to engage in healthy processes to resolve internal conflict.

“Once more of us started realizing our identities and talking with each other, [coming to terms with being transgender] was so much smoother.” (Willow System)

“For the longest time both being trans and being plural were things I suppressed… once I made that change [accepting being trans and plural] it almost made me want to cry… Plurality is just a part of my life, and it’s not anything that’s causing dysfunction. And if anything, it’s helping me live. And if I didn’t have these people, and if I tried to suppress it, I would probably be way more dysfunctional than anything.” (Orange System)

Despite living in a society that assumes cisgender and singlet experiences, valuing the authenticity of their expansive identities allowed participants to break free of expectations and focus on what truly mattered to their system. Rather than creating or compromising on a cohesive external presentation of singular identity – such as agreeing on a single set of pronouns to use with other people – some participants found that being out and proud was the best solution to meet the needs of all system members.

“To me, [being trans and plural] means breaking free from the stereotypes that I was given… when you are a system, it’s hard to be authentic because… you’re told, “You need to try to be a singlet. You need to try to conform.” And in the same way, people tell trans people, “You know, you can’t do that. You need to try to conform. If you transition, you need to be a certain way, or else you aren’t ‘doing it right.’” Being a system, that in itself is very much not conforming to the norms at all… To the outside world it may seem like, “oh, you’re always dyeing your hair. You’re always cutting it. You’re always changing your style. You’re always buying new clothes.” But to us, it’s just us being ourselves. And that feeling of like, to the outside world it’s like we’re always changing. To us, we’re just us… For us, [being trans and plural is] very much just being authentic and living the way we’ve always felt we should.” (Willow System)

## Discussion

This study analyzed transgender and plural participants’ decision-making in daily life. Three themes emerged outlining the context of conflict, decision-making processes employed, and solutions achieved by participant systems. Decisions around shared body modifications can lead to conflict between system members. However, the participant systems developed effective strategies to communicate, collaborate, achieve harmony, and make transition-related decisions with benefit to the entire group. Systems allowed individual members to express themselves, recognized each other’s needs in compromise, and practiced active internal support in dealing with conflict. Through these strategies, along with finding pride in their transgender and plural identities, participants determined how they wanted to externally present themselves and their gender identities to others. The concepts expressed in these themes can assist clinicians in all disciplines to understand experiences of trans and plural people, thereby increasing clinicians’ ability to deliver quality gender affirming medical and mental health care to this underserved population.

Participants in this study were able to effectively navigate decision-making processes related to gender transition while experiencing plurality. Trans and plural clients consistently wanted the freedom to be open with their providers about their plural identity while maintaining maximum autonomy in healthcare-related decisions once internal consensus had been reached. Our participants’ effective decision-making processes are in direct contrast to previous literature which asserted that plural systems are unable to give informed consent or make reasoned decisions; this conclusion led to the inappropriate recommendation to deny medical and surgical transition care to transgender plural clients until the multiple identities are ‘resolved’ by fusing multiple system members into a single individual [43,67–69].

The practice of withholding transition care until plural systems undergo fusion is rooted in the gatekeeping model. As described by Norton & Lindley [70], the gatekeeping model of care gives medical providers the authority to determine whether or not transgender individuals can access gender-affirming care based on their clinical judgment, which includes their biases. This results in certain kinds of trans experiences being prioritized while others are erased; many clients seeking care feel pressured to provide a dysphoria-focused, White, binary, transnormative narrative in order to avoid being denied care [70]. These effects of the gatekeeping model align with our participants’ consistent concerns about being denied transition-related healthcare if they were to be open with healthcare providers about their plural identity and related non-normative trans experiences.

Furthermore, revoking the client’s autonomy to make decisions about gender affirming care through the gatekeeping model can cause harm and medical mistrust [71]. The participant system whose psychiatrist would not authorize transition care until they had completed fusion therapy reported that they felt discouraged from seeking therapy for years. Other participants described how many of their trans and plural peers hid their plurality from healthcare providers in fear of being unable to access transition care. As clinicians, we must be careful to not unilaterally deny access to transition care based on plural identity alone, or we will continue to sow a distrust of medical providers within the trans and plural communities.

Instead, clinicians providing transition care should seek to utilize the informed consent model. The informed consent model of care encourages transgender clients to explore their experiences with a care provider, who informs them of the procedural risks and benefits before allowing the client to make an autonomous decision whether or not to engage in gender-affirming care [72,73]. While equitable access to trangender healthcare varies, when possible clinicians should move toward maximizing the autonomy of the client whether they are singlet or plural at the time of receiving care. Plural clients can be prompted to share the details of the procedure with other system members and encouraged to discuss decisions around transition care internally. Plurality is one factor, perhaps complicated, that can be explored in transition discussions, allowing systems to be more open and authentic with providers about their experiences. An understanding of the various dimensions of plurality related to communication, cooperation, internal structure, and system member dynamics can be applied in clinical settings to keep the focus on within-system communication while preserving the client’s autonomy over gender transition decisions.

The internal dynamics of being plural are often more complex than the internal dynamics of being singular [12], and many clinicians do not receive adequate training in understanding the nuance of plural experience [24,42]. Conceptualizing a plural client as a single entity accessing gender affirming care is unlikely to achieve a complete picture. Instead, clinicians can apply techniques they would use for interconnected, separate individuals, such as families or couples, to support the internal relations within the system. Plural community wisdom [74] states that the principle “As inside, so outside and vice versa” [p.1–5]. can be a useful way to understand the internal dynamics between system members; they are more like a group of people than a single individual. As such, internal relationships between system members often mirror the dynamics of interpersonal relationships between singlet (non-plural) people, including inclusive or exclusive power dynamics. Active internal care is required to nurture and maintain internal relationships between system members, just as family members and couples must continue to foster relationships over time. Internal relationship health must not be ignored within plural systems.

Clinicians not only must navigate the many internal dynamics of a plural system, but must understand how system members, both individually and as a collective, interface with the external world to appropriately work with plural decision-making. Participants often mentioned that people in their life struggled to comprehend their plurality or otherwise felt it was unsafe to be out as plural. Compromises around external presentation to prioritize social safety, including the construction of a singletsona, should not be discounted by clinicians, nor confused with fusion or progression towards singlethood. While plurals can reach a consistent agreement about their external appearance, participants noted the decisions they make to be safe within a singlet-centric society do not change their underlying experience of plurality. These decisions were reached through internal discussion, group decision-making, and often aligned with desired transition goals; clinicians should still seek to maximize clients’ decision-making autonomy in such cases.

While it can be helpful to speak directly with various system members about their relationships to gender, it may not be necessary. Participants in this study were able to report a detailed and contextual understanding of the whole system’s relationship to gender even when the interviewer only spoke to one system member. When clients have adequate internal communication, equitable internal power dynamics, and strong internal relationships, providers can gather needed information about the entire system from the member fronting at that time. Clinicians may be concerned that one system member might conceal the dissent of other members regarding gender transition. The disclosure of plurality itself is a sign of trust and openness [55]; it is important to recognize that even with singlet individuals, providers have to trust their clients to truthfully report their desire and reasoning to engage in transition-related care.

Inequitable decision-making should not be reinforced by clinicians by prioritizing or designating a single system member to make unilateral decisions, especially if done through the presumption of a singlet identity. An outside clinician granting power to one system member over the rest disempowers all of the other members of the system, as well as the system as a whole [24]. The resulting resentment and negativity towards other system members can damage internal relationships and cause further infighting. In family systems therapy, clinicians are discouraged from ‘taking sides’ with one family member and are encouraged to consider the entire family’s relationship health as ‘the client’ [75,76]. Similarly, clinicians working with plural systems should seek a neutral stance toward all system members in the decision-making process to best support the health of the whole system.

Care providers can assist trans and plural people in the decision-making process by encouraging their clients to consider the needs of all internal system members and facilitating a collaborative process to reach consensus. As with singlet individuals, clinicians should encourage plural clients to take the necessary time to explore each system member’s relationship to gender in depth; the message needs to include the assurance that taking time will not negate treatment. Conflict or ambivalence about transition should be discussed within the system until consensus between system members is reached. Clinicians can ask whether there are any further areas to explore or discussions to be held between members which would help the system come to an equitable agreement regarding transition. While they have similarities, no plural system is exactly alike. As internal dynamics, language use, experiences of gender dysphoria, and transition outcomes vary from system to system, care must be tailored to their specific experiences and treatment goals.

Clinicians can suggest helpful relationship-building techniques to systems who have not yet fostered positive internal relationships. Positive communication and collaborative efforts between system members should be supported. Clinicians can encourage system members to closely listen to one another, hold empathy for one another [12], extend warmth and acceptance to one another [39], acknowledge their vulnerability, and apologize after harming another member of the system [77]. Skills from building healthy relationships between singlets and in family therapy can be applied when building internal relationships in plural systems [78,79]. By helping the system develop loving and trusting relationships between system members, compromises can be reached about how to make changes to the body that everyone in the system can support.

Clinical flexibility is key when determining the time necessary to engage systems considering gender-affirming care. Utilizing a systems-levels approach with plural clients who have poor internal relationships, who are new to their trans identity, or who are in disagreement about transition goals may need a significant amount of time to work through the decision-making process. However, other clients may come into the office with fluid internal communication, a concrete understanding of their experiences, and easy agreement between system members. In these cases, clinical work with plurals may not take longer than working with a singlet who has already carefully thought through gender transition goals.

For plural clients who have already reached a decision about important medical choices in trans-related healthcare, clinicians should inquire about how the system came to the decision. Suggestions for lines of inquiry include asking if the decision was collectively made, which system members would be most impacted by the decision, and how individual system members experience gender. Clinicians can ask how system members communicated with one another, whether they communicated thoroughly on the topic, and how the system worked to improve communication if necessary. To assess equitable decision-making, clinicians should inquire as to whether the client had a history of dissenting opinions, and if so, ask about the process of meetings, compromises, and/or agreements utilized to resolve conflicting needs. In a similar context to exploring the potential of detransition or retransition with clients, plurals seeking transition care should be asked to explore how they would navigate discovering new system members who hold differing opinions about transition care. Collecting a client’s history of internal communication should be an exploratory process that does not create further barriers to care.

The present study lends further depth to discussion of nonlinear progress, power dynamics within the plural decision-making process, and affirming outcomes for transgender plural systems. While prior literature mentions some of the strategies illuminated here [12,33,55,56], much of it stems primarily from case studies or clinical interpretations, gives little attention to the mechanics of plural decision-making, or does not address significant and life-changing decisions. Clinicians can refer to the specific details of decision-making processes provided by the current study when working with trans and plural clients. They relate to existing frameworks with which clinicians might already be familiar, such as family systems theory. The wisdom of our participants has provided current, updated, concrete information about how trans plurals reach internal consensus and agreement. This wisdom can help providers honor their trans plural client’s experiences, understand their needs, and treat their identities and decisions with respect.

### Limitations and future research

The study has limitations. As the original interview questions did not specifically query for decision-making processes or utilize the problems-processes-solutions framework outlined in the paper, some aspects of trans plural decision making may not have been fully elaborated by participants. Because all 15 participant systems’ body ages were between 18 and 38 years, it’s unclear whether the results are transferable to older or younger populations. The inclusion criteria was participant’s self-identification as transgender and plural; only 4 out of 15 participants had received a clinical diagnosis of DID. Caution should be used when applying the results to populations that don’t identify with the terms transgender and plural; however, these results can help fill a major gap in how plurality, including DID, can be addressed in a framework that does not necessitate pathology.

Though an effort was made to reach out to diverse demographics of trans and plural people in clinical spaces accessible to the research team, recruitment was mostly through online snowball sampling. Outness to others, participation in social networks, internet access, and English language proficiency all positively factored into study participation. Moreover, participants who chose to be interviewed may disproportionately represent systems who are able to engage in internal communication processes and resolve internal conflict, in turn supporting their capacity to volunteer for the study. Recruitment from non-internet sources across multiple sites, such as behavioral health providers or in-person advocacy conferences, can help assure inclusion of under-represented subgroups without reliance upon online snowball sampling.

Further research is needed on trans and plural identities, including prevalence and health disparities for this population. Studies should assess outcomes and benefits of medical transition for the transgender and plural population, including older and younger trans plural individuals to explore the implications of transition across life history. Another significant gap in the literature involves decision-making by plurals concerning other aspects of health, education, career progress, relationships, finances, and family building. Future research is needed to delve into the ways plurals navigate other aspects of their lives and illustrate how clinicians can support plural decision-making across a variety of life contexts.

Further research on the plural population, including transgender plurals, should prioritize the involvement of community members in the conceptualization and execution of research. The present study was strengthened by the involvement of researchers with lived experience throughout the entire research process via a community participatory design.

## Supporting information

S1 Fig Interview Questions. (PDF)
