# Supplementary material for: Reaching internal consensus: Decision-making by transgender and plural people
Source: PLoS One. 2025 Oct 30;20(10):e0335714. doi: 10.1371/journal.pone.0335714 (PMC12574927; doi:10.1371/journal.pone.0335714)
Supplement: S1 Fig — (PDF) [file pone.0335714.s001.pdf]

S1 Fig. Interview Questions.

During the interview, we want to be sure to use your language. So first, we'd like to know a bit more about the language you use.

1. How do you refer to your plural group? (e.g., any combination of multiple, system, tulpamancer, person with DID, having headmates/soulbonds, endogenic, traumagenic, collective, family, or others).
2. How do you refer to the members of your group? (e.g., people, parts, alters, systemmates, headmates, tulpas)
3. Do you belong to or identify with any plural communities? More than one may apply. (e.g., DID community, healthy multiplicity community, tulpamancy community, soulbonder, daemonism). For how long?
4. What pronouns should we use to refer to you, during each interview and collectively?
5. Any other terminology you use?
6. How do you define being trans and plural for yourselves?
7. What is the intersection of experiences like for you as both trans and plural?
8. What do you like about being trans and/or plural?
9. How many other people have you told that you are plural? Trans/Gender Non-Conforming?
10. What type of support do you get (or wish you would get) from your plural community? Your trans community? People you are out to as transgender plural?
11. If you could tell the medical/psychological world one thing about being trans and plural that they need to hear, what would that be?
12. Anything else you would like to share to help others understand your experience as trans and plural people?
13. We may use a direct quote to illustrate a theme that emerges. Is there anything that you discussed that you would not like to have quoted?

(The following five questions were added for the second round of interviews)

14. What is the relationship, if any, to time spent fronting and desire to physically transition the body?

15. How do you differentiate between cisgender headmates that don't think the physical body fits them (compared to the 'body in headspace'), and trans/NB headmates that experience gender dysphoria?

16. How does gender identity impact your relationships to your body?

17. How attached are the headmates to the body?

18. Imagine you are seeking professional care regarding being transgender and plural. What are some visible ways for a professional to show they are transgender plural supportive?
